# Extracorporeal cardiopulmonary resuscitation versus conventional CPR in cardiac arrest: an updated meta-analysis and trial sequential analysis

**DOI:** 10.1186/s13054-024-04830-5

**Published:** 2024-02-21

**Authors:** Christopher Jer Wei Low, Ryan Ruiyang Ling, Kollengode Ramanathan, Ying Chen, Bram Rochwerg, Tetsuhisa Kitamura, Taku Iwami, Marcus Eng Hock Ong, Yohei Okada

**Affiliations:** 1 Yong Loo Lin School of Medicine, National University of Singapore, National Unviersity Health System, Singapore, Singapore; 2 Cardiothoracic Intensive Care Unit, National University Heart Centre Singapore, National University Health System, Singapore, Singapore; 3 Genome Institute of Singapore, Agency for Science, Technology and Research (A*STAR), Singapore, Singapore; 4 Division of Critical Care, Department of Medicine, McMaster University, Hamilton, ON Canada; 5 Department of Health Research Methods, Evidence, and Impact, McMaster University, Hamilton, ON Canada; 6 Department of Social and Environmental Medicine, Graduate School of Medicine, Osaka University, Osaka, Japan; 7 Preventive Services, Graduate School of Medicine, School of Public Health, Kyoto University, Kyoto, Japan; 8 Health Services and Systems Research, Duke-NUS Medical School, Singapore, Singapore; 9 Department of Emergency Medicine, Singapore General Hospital, Singapore, Singapore

**Keywords:** Extracorporeal membrane oxygenation, Cardiac arrest, Cardiopulmonary resuscitation, Meta-analysis

## Abstract

**Background:**

Extracorporeal cardiopulmonary resuscitation (ECPR) may reduce mortality and improve neurological outcomes in patients with cardiac arrest. We updated our existing meta-analysis and trial sequential analysis to further evaluate ECPR compared to conventional CPR (CCPR).

**Methods:**

We searched three international databases from 1 January 2000 through 1 November 2023, for randomised controlled trials or propensity score matched studies (PSMs) comparing ECPR to CCPR in both out-of-hospital cardiac arrest (OHCA) and in-hospital cardiac arrest (IHCA). We conducted an updated random-effects meta-analysis, with the primary outcome being in-hospital mortality. Secondary outcomes included short- and long-term favourable neurological outcome and survival (30 days–1 year). We also conducted a trial sequential analysis to evaluate the required information size in the meta-analysis to detect a clinically relevant reduction in mortality.

**Results:**

We included 13 studies with 14 pairwise comparisons (6336 ECPR and 7712 CCPR) in our updated meta-analysis. ECPR was associated with greater precision in reducing overall in-hospital mortality (OR 0.63, 95% CI 0.50–0.79, high certainty), to which the trial sequential analysis was concordant. The addition of recent studies revealed a newly significant decrease in mortality in OHCA (OR 0.62, 95% CI 0.45–0.84). Re-analysis of relevant secondary outcomes reaffirmed our initial findings of favourable short-term neurological outcomes and survival up to 30 days. Estimates for long-term neurological outcome and 90-day–1-year survival remained unchanged.

**Conclusions:**

We found that ECPR reduces in-hospital mortality, improves neurological outcome, and 30-day survival. We additionally found a newly significant benefit in OHCA, suggesting that ECPR may be considered in both IHCA and OHCA.

**Supplementary Information:**

The online version contains supplementary material available at 10.1186/s13054-024-04830-5.

## Introduction

Despite advances in research, prognosis following cardiac arrest remains grim [1, 2]. Extracorporeal cardiopulmonary resuscitation (ECPR) in refractory cardiac arrest can potentially be considered, yet the utility of ECPR in out-of-hospital cardiac arrest (OHCA) remains to be seen, with three randomised controlled trials (RCTs) reporting differing outcomes [3–6]. Our group recently conducted a systematic review and meta-analysis of randomised clinical trials (RCTs) and propensity score matched studies on ECPR in cardiac arrest [7]. While we found a reduction in mortality with ECPR for in-hospital cardiac arrest (IHCA), we did not observe this same finding in OHCA, although this latter conclusion may have been impacted by ongoing imprecision. We concluded that more studies were needed assessing the role of ECPR, specifically in OHCA.

The publication of a new propensity score matched study by Okada et al. [8] evaluating ECPR in over 2000 patients with OHCA is a timely addition to the literature, along with a similar study by Choi et al. [9]. We believe that the publication of these new studies stands to enhance our understanding of this topic. As the evidence has expanded, we updated the systematic review and meta-analysis based on our previous study to evaluate the ECPR among OHCA patients.

## Methods

The original protocol was registered with PROSPERO (CRD42022332623). We adhered to the Preferred Reporting Items for Systematic reviews and Meta-Analysis (PRISMA) statement (Additional file 1: PRISMA Checklist) [10], and the prespecified methodology and analytical plan from our original meta-analysis. Briefly, we updated our literature search and screened the literature through 1 November 2023. We followed the prior inclusion criteria of either RCTs or propensity score matched studies comparing ECPR against conventional cardiopulmonary resuscitation (CCPR) in cardiac arrest. We did a random-effects meta-analyses (Mantel–Haenszel method) of binary outcomes using the DerSimonian–Laird model [11, 12] and present outcomes as pooled odds ratios (OR) with 95% confidence intervals (CIs). We adhered to the Grading of Recommendations, Assessment, Development, and Evaluations approach when assessing the certainty of evidence [13]. We assessed risk of bias via the Cochrane Risk of Bias 2 Tool for RCTs and Newcastle Ottawa Score for PSMs.

The primary outcome was in-hospital mortality. We also re-analysed relevant secondary outcomes where reported by any of the newly included studies. These were favourable short-term (discharge to 30 days) neurological outcome (defined by a cerebral performance category (CPC) score of 1–2), as well as post-discharge survival of 30 days. We repeated the prespecified subgroup analyses and the trial sequential analysis.

Further details on the original methodology, along with their references, can be found in Additional file 1: Original Methods and References for Original Methods. As both shockable and non-shockable cohorts in Okada et al. [8] were matched separately, we have included these cohorts separately in this updated analysis. We used p-values of less than 0.05 as the threshold for statistical significance. We did all meta-analyses analyses using R (version 4.0.5) and trial sequential analyses using TSA v0.9.5.10 (www.ctu.dk/tsa).

## Results

### Updated primary outcomes

We updated our search until 1 November 2023, including 13 studies (three RCTs and 10 PSMs) with 14 pairwise comparisons comprising 14,048 patients (6336 ECPR and 7712 CCPR, references in Additional file 1: PRISMA Flowchart and References for Included Studies). Study characteristics for individual studies are noted in Table 1. All RCTs were noted to be either at ‘low risk’ or ‘some concerns’ for bias, while all PSMs were noted to be of ‘high quality’ (Additional file 1: Table S1a and S1b). ECPR was associated with lower mortality (OR 0.63, 95% CI 0.50–0.79), with high certainty based on GRADE (Additional file 1: Table S2). Due to concerns regarding possible overlaps in Korean OHCA data [14], we conducted sensitivity analysis excluding Choi et al. [9]; the pooled estimate did not substantially change.

**Table 1:** General characteristics of all included studies

Study	Study type	Region	Location of arrest	Intervention	Sample size/persons	Age/years	Initial presenting rhythm/persons	In-hospital mortality/ persons	30-day survival/ persons	Short-term favourable neurological outcome /persons
Belohlavek (2022) [6]	RCT	Europe	Out-of-hospital	ECPR	124	59 ± 13.5	72 VF 31 asystole 21 PEA	84	52	38
				CCPR	132	57 ± 13.49	84 VF 24 asystole 24 PEA	101	43	24
Blumenstein (2016)[15]	PSM	Europe	In-hospital	ECPR	52	72 ± 17.46	32 sinus without AV block 15 atrial fibrillation 1 AV block III 1 VT 3 unspecified	38	14	11
				CCPR	52	73 ± 7.62	29 sinus without AV block 13 atrial fibrillation 3 AV block III 3 PEA 2 VT 2 asystole	43	9	7
Chen (2008)[16]	PSM	Asia	In-hospital	ECPR	46	57 ± 14	21 VT/VF 15 PEA 10 asystole	31	16	14
				CCPR	46	55 ± 15	19 VT/VF 18 PEA 9 asystole	38	8	7
Choi (2023) [9]	PSM	Asia	Out-of-hospital	ECPR	458	55.5 ± 14.1	271 VT/VF 96 PEA 91 asystole	391	N/A	47
				CCPR	1832	56.4 ± 16.1	1052 VT/VF 394 PEA 386 asystole	1578	N/A	127
Jeong (2022) [17]	PSM	Asia	Out-of-hospital	ECPR	271	58.0 ± 13.4	N/A	226	45	35
				CCPR	271	58.3 ± 17.9		218	53	44
Kim (2020) [14]	PSM	Asia	Out-of-hospital	ECPR	3826	59.8 ± 14.9	N/A	2873	953	N/A
				CCPR	3826	59.7 ± 15.1		3080	746	N/A
Lin (2010) [18]	PSM	Asia	In-hospital	ECPR	27	59 ± 11	15 VT/VF 7 PEA 5 asystole	19	9	7
				CCPR	27	60 ± 13	18 VT/VF 6 PEA 3 asystole	22	7	5
Maekawa (2013) [19]	PSM	Asia	Out-of-hospital	ECPR	24	57 ± 11.82	13 VT/VF	15	N/A	N/A
				CCPR	24	57 ± 14.19	14 VT/VF	21	N/A	N/A
Okada (2023) [8] (shockable rhythm cohort)	PSM	Asia	Out-of-hospital	ECPR	913	58.3 ± 14.9	913 VT/VF	688*	225	109
				CCPR	913	59.0 ± 16.3	913 VT/VF	764*	149	100
Okada (2023) [8] (non-shockable rhythm cohort)	PSM	Asia	Out-of-hospital	ECPR	370	58.7 ± 16.4	237 PEA 133 asystole	326*	44	17
				CCPR	370	57.3 ± 17.9	239 PEA 131 asystole	361*	9	4
Patricio (2019) [20]	PSM	Europe	Both in- and out-of-hospital	ECPR	80	57 ± 14	24 VF/VT 35 asystole 21 PEA	62	N/A	N/A
				CCPR	80	57 ± 17	23 VT/VF 38 asystole 19 PEA	66	N/A	N/A
Shin (2011/13) [23, 24]	PSM	Asia	Out-of-hospital	ECPR	60	60.8 ± 14.5	13 VT/VF 41 PEA 6 asystole	41	19	14
				CCPR	60	60.5 ± 15.5	13 VT/VF 41 PEA 6 asystole	54	6	3
Suverein (2023) [4]	RCT	Europe	Out-of-hospital	ECPR	70	54 ± 12	69 VT/VF/PEA 11 secondary arrhythmia 1 Pulmonary embolism	56	N/A	14
				CCPR	64	57 ± 10	63 VT/VF/PEA 11 secondary arrhythmia 0 pulmonary embolism	51	N/A	10
Yannopoulos (2020) [5]	RCT	North America	Out-of-hospital	ECPR	15	59 ± 10	15 VF	9	N/A	N/A
				CCPR	15	58 ± 11	15 VF	14	N/A	N/A

*AMI* acute myocardial infarction, *AV* atrioventricular, *AVR* aortic valve replacement, *CABG* coronary artery bypass graft, *CAD* coronary artery disease, *CCPR* conventional cardiopulmonary resuscitation, *ECPR* extracorporeal cardiopulmonary resuscitation, *HF* heart failure, *ICD* implantable cardiac defibrillator, *LVEF* left ventricular ejection fraction, *MVR* mitral valve replacement, *N/A* not available, *PCI* percutaneous coronary intervention, *PEA* pulse electrical activity, *PSM* propensity score matched study, *RCT* randomised controlled trial, *VF* ventricular fibrillation, and *VT* ventricular tachycardia. In-hospital mortality is imputed by 30-day survival

**Fig. 1:**
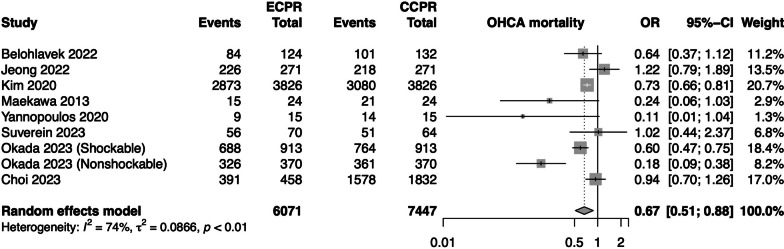
Forest plot for OHCA. CCPR, conventional cardiopulmonary resuscitation, ECPR, extracorporeal cardiopulmonary resuscitation, OHCA, out-of-hospital cardiac arrest, OR, odds ratio, and CI, confidence interval

Subgroup analysis examining mortality based on type of study, geographical region, location of arrest (OHCA vs in-hospital cardiac arrest [IHCA]), and study quality did not demonstrate significant differences (*p*_interaction_ > 0.05 for all subgroup comparisons). As compared to the previous analysis, the overall findings were very similar. However, for patients with OHCA, while ECPR previously had no association with mortality reduction, the updated analysis, including these two new studies, now demonstrated a reduction in mortality with ECPR (OR 0.67, 95% CI 0.51–0.88, Fig. 1). Trial sequential analysis for mortality found that the cumulative *Z*-curve passed the required information size and TSA-adjusted boundary for benefit affirmed our results.

### Updated secondary outcomes

Updated analysis of relevant secondary outcomes found that ECPR was associated with favourable neurological outcomes at short-term follow-up (OR 1.57, 95% CI 1.14–2.15, high certainty) and survival up to 30 days (OR 1.70, 95% CI 1.29–2.26, high certainty), for which trial sequential analyses demonstrated that required information size was met, and TSA-adjusted boundaries were consistent. These findings were both similar to what we had previously demonstrated.

Additional file 1: Table S3 summarises these updated findings for all updated analyses, including subgroup analysis. While reported in our previously published meta-analysis, there were no new data evaluating long-term (3 months, 6 months, and 1 year) survival and long-term neurological outcomes in the newly included studies. As such, these outcomes were not updated and presented in this paper. These outcomes can be found in the initial report [7]. We have also indicated the result of post hoc sensitivity analysis in Additional file 1: Table S4.

## Discussion

The most pertinent change in findings from this updated analysis were improvements in mortality when using ECPR for patients with OHCA, which represents a change from our previously published results [7]. This change in findings is likely a result of improvements in precision achieved with including additional studies and therefore larger number of patients and events.

Although both new studies had large sample sizes, it was Okada et al. [8] which included both shockable and non-shockable cohorts that primarily led to improvements in precision and generalisability of results, and an improved estimate in the OHCA cohort. The other new study, Choi et al. [9], did not see any significant differences in mortality with ECPR, with results being imprecise.

In our previously published report, we discussed differing conclusions between the three RCTs examining this topic and proposed that varying times to cannulation between these studies might explain their differing results, with the RCT having the shortest time to cannulation having the greatest benefit seen in ECPR. We posit that this same variable may also explain the shift in these updated results, particularly with reference to the inclusion of Okada et al. [8]. Okada and colleagues were able to achieve extremely fast time to ECPR cannulation, with a median time of under 30 min in both shockable and non-shockable cohorts, which corroborates with the findings from our prior meta-regression that mortality increases with low-flow time (HR [hazard ratio] per min: 1.01, 95% CI 1.00–1.01). In Japan, ECPR is often initiated at the emergency department, without the need to involve inpatient services, unlike in other regions [21]. This would result in faster cannulation than if the patient had to be transported to the operating theatre or other procedural rooms in order to achieve cannulation. This decrease in low-flow time likely reduces multiorgan failure and brain injury after cardiac arrest, thereby augmenting the survival benefits of ECPR [3, 22].

These studies supplement our prior analysis, with the addition of additional OHCA cohorts now demonstrating the benefit of ECPR in both OHCA and IHCA. Nevertheless, factors such as the preparedness of pre-hospital ECMO programmes and the speed at which ECMO cannulation is achieved affect outcomes, and it remains a highly labour- and resource-intense intervention. ECPR can only be as effective as the team that is providing it, with effectiveness likely only seen in high-volume centres able to achieve expeditious cannulation.

This study has limitations that should be considered. Importantly, residual confounding remains an issue in PSMs, with factors outside the propensity model not accounted for and potentially confounding the analysis. Furthermore, there are no clear, unified eligibility criteria for ECPR. Each study in this meta-analysis reported variable selection criteria, which necessitates a cautious interpretation of the results. Furthermore, it is noted that among the RCTs included in this analysis, two were terminated early and did not reach the pre-defined sample size [5, 6], one had a number of post-randomisation exclusions [4], and others had protocolised features that may have impacted maintenance of blinding [4, 5]. Also, there is some variability in our risk of bias assessments and those of previously published systematic reviews, and while we are confident in our assessments (Additional file 1: Table S1a and S1b), this highlights the subjectivity that can be associated with these ratings [25]. Additionally, we used mortality as the primary outcome, which although patient important, does not provide information related to quality of life. This was prespecified in our protocol, and based on the fact that we thought more of the included studies would report on this endpoint, that being said, we have included survival with favourable neurological outcome as a secondary outcome in the analysis.

## Conclusion

In conclusion, we found that ECPR was associated with significant reductions in mortality for patients with cardiac arrest, with a significant reduction in patients with OHCA upon addition of new studies. Compared with CCPR, ECPR also improves short-term neurological outcomes and 30-day survival, affirming our prior analysis.

## Supplementary Information

**Additional file 1**. PRISMA Checklist. Original Methods and References for Original Method. PRISMA Flowchart and References for Included Studies.

## Data Availability

All data generated or analysed in this study were extracted from published studies and their supplementary information files.
