# Supplementary material for: Extracorporeal cardiopulmonary resuscitation versus conventional CPR in cardiac arrest: an updated meta-analysis and trial sequential analysis
Source: Crit Care. 2024 Feb 21;28:57. doi: 10.1186/s13054-024-04830-5 (PMC10882798; doi:10.1186/s13054-024-04830-5)
Supplement: Supplementary file 1 — Additional file 1. PRISMA Checklist. Original Methods and References for Original Method. PRISMA Flowchart and References for Included Studies. [file 13054_2024_4830_MOESM1_ESM.docx]

**Extracorporeal cardiopulmonary resuscitation versus conventional CPR in cardiac arrest: an updated meta-analysis and trial sequential analysis**

***Supplementary Appendix***

**Table of Contents**

[PRISMA Flowchart １](#_Toc158923850)

[PRISMA Checklist 1](#_Toc158923851)

[Original Methods 4](#_Toc158923852)

[**Search strategy and selection criteria 4**](#_Toc158923853)

[**Data collection and risk of bias assessment 4**](#_Toc158923854)

[**Data synthesis 4**](#_Toc158923855)

[**Post hoc analysis 5**](#_Toc158923856)

[References for Original Methods 6](#_Toc158923857)

[References for Included Studies 7](#_Toc158923858)

[Table S1a. Cochrane risk of bias tool 2.0 for randomised controlled trials 8](#_Toc158923859)

[Table S1b. Newcastle Ottawa Score for cohort studies 8](#_Toc158923860)

[Table S2. Grading of Recommendations, Assessment, Development, and Evaluations 9](#_Toc158923861)

[Table S3: Overall table summarising the re-analysed results, with forest plots and trial-sequential analyses graphs 10](#_Toc158923862)

**[Table S4: Post-hoc sensitivity analysis 14](#_Toc158923863)**

# **PRISMA Flowchart**


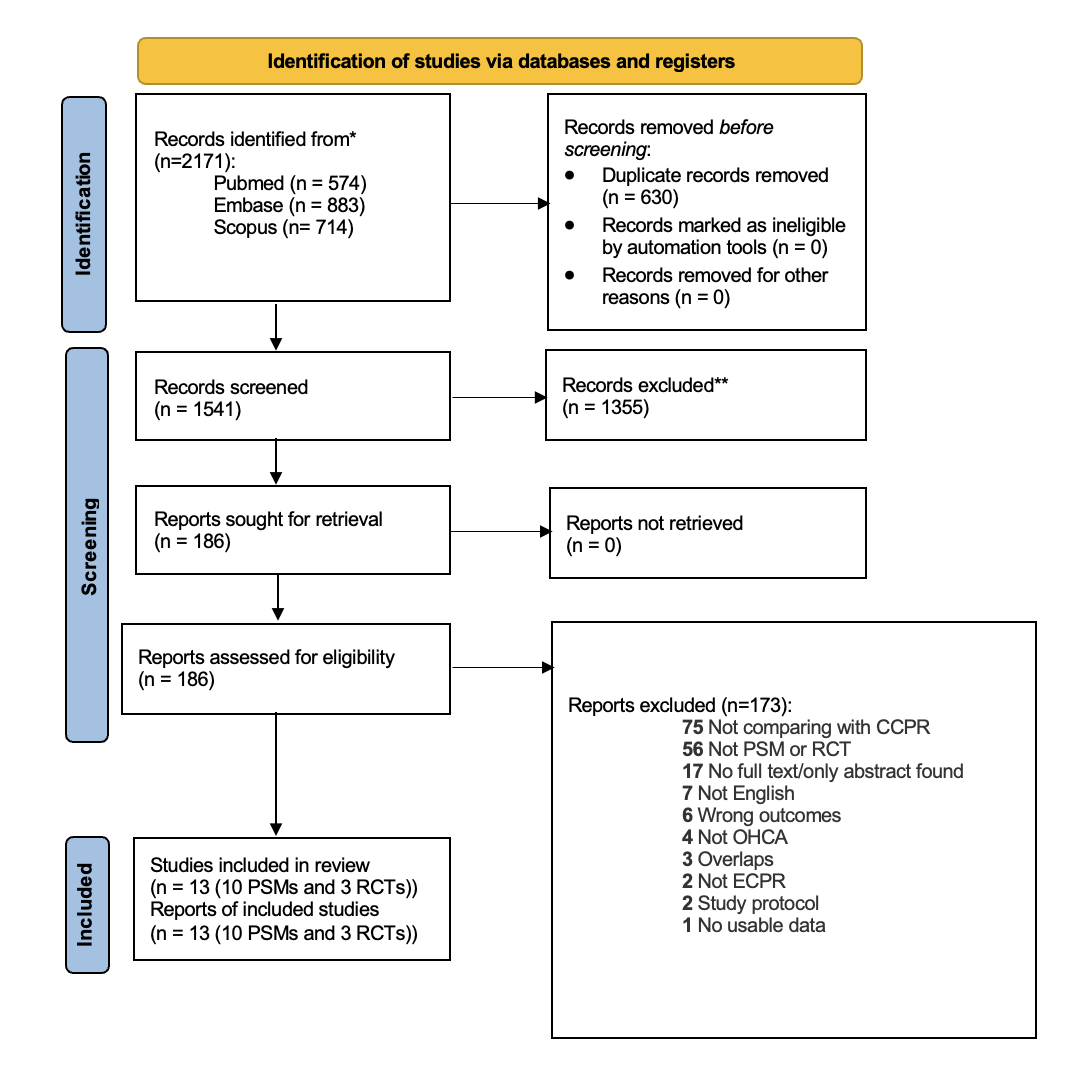


*From:*  Page MJ, McKenzie JE, Bossuyt PM, Boutron I, Hoffmann TC, Mulrow CD, et al. The PRISMA 2020 statement: an updated guideline for reporting systematic reviews. BMJ 2021;372:n71. doi: 10.1136/bmj.n71

# **PRISMA Checklist**

| **Section and Topic** | **Item #** | **Checklist item** | **Location where item is reported** |
| --- | --- | --- | --- |
| **TITLE** | | |  |
| Title | 1 | Identify the report as a systematic review. | 3 |
| **ABSTRACT** | | |  |
| Abstract | 2 | See the PRISMA 2020 for Abstracts checklist. | 3 |
| **INTRODUCTION** | | |  |
| Rationale | 3 | Describe the rationale for the review in the context of existing knowledge. | 5 |
| Objectives | 4 | Provide an explicit statement of the objective(s) or question(s) the review addresses. | 5 |
| **METHODS** | | |  |
| Eligibility criteria | 5 | Specify the inclusion and exclusion criteria for the review and how studies were grouped for the syntheses. | 5-6, Supplementary data, Original Methods |
| Information sources | 6 | Specify all databases, registers, websites, organisations, reference lists and other sources searched or consulted to identify studies. Specify the date when each source was last searched or consulted. | 5-6, Supplementary data: Original Methods |
| Search strategy | 7 | Present the full search strategies for all databases, registers and websites, including any filters and limits used. | 5-6, Supplementary data: Original Methods |
| Selection process | 8 | Specify the methods used to decide whether a study met the inclusion criteria of the review, including how many reviewers screened each record and each report retrieved, whether they worked independently, and if applicable, details of automation tools used in the process. | 5-6, Supplementary data: Original Methods |
| Data collection process | 9 | Specify the methods used to collect data from reports, including how many reviewers collected data from each report, whether they worked independently, any processes for obtaining or confirming data from study investigators, and if applicable, details of automation tools used in the process. | 5-6, Supplementary data: Original Methods |
| Data items | 10a | List and define all outcomes for which data were sought. Specify whether all results that were compatible with each outcome domain in each study were sought (e.g. for all measures, time points, analyses), and if not, the methods used to decide which results to collect. | 5-6, Supplementary data: Original Methods |
|  | 10b | List and define all other variables for which data were sought (e.g. participant and intervention characteristics, funding sources). Describe any assumptions made about any missing or unclear information. | 5-6, Supplementary data: Original Methods |
| Study risk of bias assessment | 11 | Specify the methods used to assess risk of bias in the included studies, including details of the tool(s) used, how many reviewers assessed each study and whether they worked independently, and if applicable, details of automation tools used in the process. | 6, Supplementary data: Original Methods |
| Effect measures | 12 | Specify for each outcome the effect measure(s) (e.g. risk ratio, mean difference) used in the synthesis or presentation of results. | 6, Supplementary data: Original Methods |
| Synthesis methods | 13a | Describe the processes used to decide which studies were eligible for each synthesis (e.g. tabulating the study intervention characteristics and comparing against the planned groups for each synthesis (item #5)). | 6, Supplementary data: Original Methods |
|  | 13b | Describe any methods required to prepare the data for presentation or synthesis, such as handling of missing summary statistics, or data conversions. | 6, Supplementary data: Original Methods |
|  | 13c | Describe any methods used to tabulate or visually display results of individual studies and syntheses. | 6, Supplementary data: Original Methods |
|  | 13d | Describe any methods used to synthesize results and provide a rationale for the choice(s). If meta-analysis was performed, describe the model(s), method(s) to identify the presence and extent of statistical heterogeneity, and software package(s) used. | 6, Supplementary data: Original Methods |
|  | 13e | Describe any methods used to explore possible causes of heterogeneity among study results (e.g. subgroup analysis, meta-regression). | 6, Supplementary data: Original Methods |
|  | 13f | Describe any sensitivity analyses conducted to assess robustness of the synthesized results. | 6, Supplementary data: Original Methods |
| Reporting bias assessment | 14 | Describe any methods used to assess risk of bias due to missing results in a synthesis (arising from reporting biases). | 6, Supplementary data: Original Methods |
| Certainty assessment | 15 | Describe any methods used to assess certainty (or confidence) in the body of evidence for an outcome. | 6, Supplementary data: Original Methods |
| **RESULTS** | | |  |
| Study selection | 16a | Describe the results of the search and selection process, from the number of records identified in the search to the number of studies included in the review, ideally using a flow diagram. | 6 |
|  | 16b | Cite studies that might appear to meet the inclusion criteria, but which were excluded, and explain why they were excluded. | 6 |
| Study characteristics | 17 | Cite each included study and present its characteristics. | 6, Supplementary data: References for included studies |
| Risk of bias in studies | 18 | Present assessments of risk of bias for each included study. | Supplementary data: Table S1 |
| Results of individual studies | 19 | For all outcomes, present, for each study: (a) summary statistics for each group (where appropriate) and (b) an effect estimate and its precision (e.g. confidence/credible interval), ideally using structured tables or plots. | 7, Supplementary data: Table S3 |
| Results of syntheses | 20a | For each synthesis, briefly summarise the characteristics and risk of bias among contributing studies. | 7, Supplementary data: Table S1 |
|  | 20b | Present results of all statistical syntheses conducted. If meta-analysis was done, present for each the summary estimate and its precision (e.g. confidence/credible interval) and measures of statistical heterogeneity. If comparing groups, describe the direction of the effect. | 7, Supplementary data: Table S3 |
|  | 20c | Present results of all investigations of possible causes of heterogeneity among study results. | 7, Supplementary data: Table S3 |
|  | 20d | Present results of all sensitivity analyses conducted to assess the robustness of the synthesized results. | 6-7 |
| Reporting biases | 21 | Present assessments of risk of bias due to missing results (arising from reporting biases) for each synthesis assessed. | Supplementary data: Table S1 |
| Certainty of evidence | 22 | Present assessments of certainty (or confidence) in the body of evidence for each outcome assessed. | Supplementary data: Table S2 |
| **DISCUSSION** | | |  |
| Discussion | 23a | Provide a general interpretation of the results in the context of other evidence. | 8-9 |
|  | 23b | Discuss any limitations of the evidence included in the review. | 9 |
|  | 23c | Discuss any limitations of the review processes used. | 9 |
|  | 23d | Discuss implications of the results for practice, policy, and future research. | 9-10 |
| **OTHER INFORMATION** | | |  |
| Registration and protocol | 24a | Provide registration information for the review, including register name and registration number, or state that the review was not registered. | 5 |
|  | 24b | Indicate where the review protocol can be accessed, or state that a protocol was not prepared. | 5 |
|  | 24c | Describe and explain any amendments to information provided at registration or in the protocol. | 5 |
| Support | 25 | Describe sources of financial or non-financial support for the review, and the role of the funders or sponsors in the review. | 11-12 |
| Competing interests | 26 | Declare any competing interests of review authors. | 11-12 |
| Availability of data, code and other materials | 27 | Report which of the following are publicly available and where they can be found: template data collection forms; data extracted from included studies; data used for all analyses; analytic code; any other materials used in the review. | 11-12 |

#

#

#

#

#

#

#

#

#

#

#

#

#

#

#

#

#

#

#

# **Original Methods**

### *Search strategy and selection criteria*

The protocol was registered with PROSPERO (CRD42022332623), which can be found online at the link (https://www.crd.york.ac.uk/prospero/display_record.php?RecordID=332623). The review conducted in adherence with the Preferred Reporting Items for Systematic Reviews and Meta-analyses (PRISMA) Statement.[1]. MEDLINE, Embase and Scopus databases were searched from January 1, 2000, to April 1, 2023, using the following keywords and their variations: “extracorporeal membrane oxygenation”, “cardiopulmonary resuscitation” or “cardiac arrest”, and “randomised controlled trial” or “propensity”. Reference lists of included studies and prior review articles were also included. Only RCTs and PSMs comparing ECPR with CCPR in adults with cardiac arrest were included, excluding any studies reporting on patients <18 years old or non-human studies, and any other observational studies. In the case of overlapping patient data, only the largest study was included.

### *Data collection and risk of bias assessment*

A prespecified data extraction form was used to collect data. The Cochrane Risk of Bias 2.0 tool for RCTs and the Newcastle Ottawa Scale (NOS) for cohort studies were used to assess risk of bias, and the Grading of Recommendations, Assessments, Developments and Evaluations (GRADE) approach used to assess certainty of evidence. The GRADE approach ranks the certainty of evidence from “high” (true effect likely lies close to that of the estimate of the effect) to “very low” (true effect is likely to be substantially different from the estimate of effect).[2, 3] CJWL, MJCH, and RRL screened the studies independently and in duplicate, collected the data, and assessed the risk of bias. Conflicts were resolved by KR. Corresponding authors of each study were contacted to obtain additional data for analysis where necessary.

### *Data synthesis*

In-hospital mortality was chosen as the primary outcome. Secondary outcomes included favourable neurological outcome (defined by a Cerebral Performance Category (CPC) score of 1-2) in the short-term (discharge to 30 days) and long-term (≥90 days), and post-discharge survival (30 days, 3 months, 6 months, and 1 year) and complications during ECMO (classified in broad groups described by the Extracorporeal Life Support Organisation [ELSO]. Random-effects meta-analyses (Mantel and Haenszel) was conducted for binary outcomes using the DerSimonian and Laird model, [4-6] and conventional inverse-variance weighted meta-analyses for continuous outcomes.[7] Binary outcomes are presented as pooled odds ratios (OR), and continuous outcomes as mean differences, each with corresponding 95% confidence intervals (CIs). Statistical heterogeneity (inconsistency) as part of the GRADE approach was assessed quantitatively using I-squared but also tau-squared values and p-values from the Cochran Q test, and qualitatively via visual inspection of forest plots.[8] Publication bias was qualitatively assessed using visual inspection of funnel plots, and quantitatively via Egger’s regression test. Small-study effects were corrected for using the random-effects trim-and-fill (R_0_ estimator) procedure, and a sensitivity analysis excluding studies noted to be at high risks of bias (Cochrane Risk of Bias 2.0 “high” risk, or NOS score <7) was also conducted.

Prespecified subgroup analysis was conducted based on location of arrest (in-hospital or out of hospital), study type (PSM or RCT), study quality, and geographical region (Asia, Europe, North America). When at least 6 data points were reported, random-effects inverse variance[9] univariable meta-regression was conducted to explore potential sources of heterogeneity, or prognostically relevant prespecified study-level covariates (centre volume [per doubling of centre volume], age [per year], proportion of male patients, body mass index [per 1 kg/m^2^], duration of CPR [minutes] and proportion of patients presenting with ventricular fibrillation or tachycardia). Centre volume in observational studies was obtained by dividing the number of patients who received ECPR by the number of centres, and the number of years comprising the study period. Centre volume in RCTs was derived via the number of patients who were eligible for ECPR. Previous reviews of the ELSO registry have reported similar methods for calculating centre volume. [10, 11]

As additional analysis, trial sequential analysis (TSA) using TSA v0.9.5.10 (http://www.ctu.dk/tsa), was conducted, assessing efficacy based on the O’Brien-Fleming alpha-spending function, and futility based on the beta-spending function. A TSA combines results of studies in a meta-analysis by conducting a sample size calculation to evaluate a cumulative pooled effect after each additional trial is included based on the information size thus obtained. This is similar to group sequential monitoring boundaries in RCTs during interim analyses.[12] Required information size (RIS) and cumulative Z-scores were estimated using the relative risk reduction and baseline estimates of the conventional CPR group from the results of the meta-analysis. Variance of the pooled estimate and heterogeneity were estimated using the TSA software, with an assumed type I error of 5% and a power of 80%. For continuous variables, the means from the aggregate data presented in each study as per Wan et al were pooled.[13] A *p* value of < 0.05 was defined as statistically significant, all statistical analyses were performed using R 4.0.5.

### *Post hoc analysis*

In order to adjust for potential factors that might confound the association between mortality with ECPR, adjusted ORs and hazard ratios (HRs) were pooled. In addition, HRs for mortality for potential prognostic factors, including age (per year), duration of CPR (per minute), and initial presenting rhythm (shockable vs unshockable) were also pooled to better understand certain prognostic factors which might affect mortality. Additional post-hoc analysis including all observational studies that did not undertake propensity-score matching was also conducted.

Furthermore, we have conducted an additional sensitivity analysis estimating the differences in survival to hospital discharge (the analogue of in-hospital mortality), and evaluating the robustness of the main results using the data from the studies with low risk on ROB2 and studies scoring 9 on NOS.

# **References for Original Methods**

1. Page MJ, McKenzie JE, Bossuyt PM, Boutron I, Hoffmann TC, Mulrow CD, Shamseer L, Tetzlaff JM, Akl EA, Brennan SE *et al*: **The PRISMA 2020 statement: an updated guideline for reporting systematic reviews**. *Bmj* 2021, **372**:n71.

2. Guyatt G, Oxman AD, Akl EA, Kunz R, Vist G, Brozek J, Norris S, Falck-Ytter Y, Glasziou P, DeBeer H *et al*: **GRADE guidelines: 1. Introduction-GRADE evidence profiles and summary of findings tables**. *J Clin Epidemiol* 2011, **64**(4):383-394.

3. Balshem H, Helfand M, Schünemann HJ, Oxman AD, Kunz R, Brozek J, Vist GE, Falck-Ytter Y, Meerpohl J, Norris S *et al*: **GRADE guidelines: 3. Rating the quality of evidence**. *J Clin Epidemiol* 2011, **64**(4):401-406.

4. Greenland S, Robins JM: **Estimation of a common effect parameter from sparse follow-up data**. *Biometrics* 1985, **41**(1):55-68.

5. Robins J, Breslow N, Greenland S: **Estimators of the Mantel-Haenszel variance consistent in both sparse data and large-strata limiting models**. *Biometrics* 1986, **42**(2):311-323.

6. DerSimonian R, Laird N: **Meta-analysis in clinical trials**. *Controlled Clinical Trials* 1986, **7**(3):177-188.

7. Balduzzi S, Rücker G, Schwarzer G: **How to perform a meta-analysis with R: a practical tutorial**. *Evid Based Ment Health* 2019, **22**(4):153-160.

8. Guyatt GH, Oxman AD, Kunz R, Woodcock J, Brozek J, Helfand M, Alonso-Coello P, Glasziou P, Jaeschke R, Akl EA *et al*: **GRADE guidelines: 7. Rating the quality of evidence--inconsistency**. *J Clin Epidemiol* 2011, **64**(12):1294-1302.

9. Borenstein M, Hedges LV, Higgins JP, Rothstein HR: **A basic introduction to fixed-effect and random-effects models for meta-analysis**. *Res Synth Methods* 2010, **1**(2):97-111.

10. Barbaro RP, MacLaren G, Boonstra PS, Combes A, Agerstrand C, Annich G, Diaz R, Fan E, Hryniewicz K, Lorusso R *et al*: **Extracorporeal membrane oxygenation for COVID-19: evolving outcomes from the international Extracorporeal Life Support Organization Registry**. *Lancet* 2021, **398**(10307):1230-1238.

11. Barbaro RP, MacLaren G, Boonstra PS, Iwashyna TJ, Slutsky AS, Fan E, Bartlett RH, Tonna JE, Hyslop R, Fanning JJ *et al*: **Extracorporeal membrane oxygenation support in COVID-19: an international cohort study of the Extracorporeal Life Support Organization registry**. *Lancet* 2020, **396**(10257):1071-1078.

12. Ling RR, Sim JJL, Tan FL, Tai BC, Syn N, Mucheli SS, Fan BE, Mitra S, Ramanathan K: **Convalescent Plasma for Patients Hospitalized With Coronavirus Disease 2019: A Meta-Analysis With Trial Sequential Analysis of Randomized Controlled Trials**. *Transfus Med Rev* 2022, **36**(1):16-26.

13. Wan X, Wang W, Liu J, Tong T: **Estimating the sample mean and standard deviation from the sample size, median, range and/or interquartile range**. *BMC Medical Research Methodology* 2014, **14**(1):135.

# **References for Included Studies**

1. Belohlavek J, Smalcova J, Rob D, et al. **Effect of intra-arrest transport, extracorporeal cardiopulmonary resuscitation, and immediate invasive assessment and treatment on functional neurologic outcome in refractory out-of-hospital cardiac arrest: a randomized clinical trial.** *JAMA* 2022; **327:** 737–47.
2. Blumenstein J, Leick J, Liebetrau C, et al. **Extracorporeal life support in cardiovascular patients with observed refractory in-hospital cardiac arrest is associated with favourable short and long-term outcomes: a propensity-matched analysis.** *Eur Heart J Acute Cardiovasc Care* 2016; **5:** 13–22.
3. Chen Y-S, Lin J-W, Yu H-Y, et al. **Cardiopulmonary resuscitation with assisted extracorporeal life-support versus conventional cardiopulmonary resuscitation in adults with in-hospital cardiac arrest: an observational study and propensity analysis.** *Lancet* 2008; **372:** 554–61.
4. Choi Y, Park JH, Jeong J, Kim YJ, Song KJ, Shin SD: **Extracorporeal cardiopulmonary resuscitation for adult out-of-hospital cardiac arrest patients: time-dependent propensity score-sequential matching analysis from a nationwide population-based registry**. *Critical Care* 2023, **27**(1):87.
5. Jeong D, Lee GT, Park JE, et al. **Extracorporeal life-support for out-of-hospital cardiac arrest: a nationwide multicenter study.** *Shock* 2022; **57:** 680–86.
6. Kim SJ, Han KS, Lee EJ, Lee SJ, Lee JS, Lee SW. **Association between extracorporeal membrane oxygenation (ECMO) and mortality in the patients with cardiac arrest: a nation-wide population-based study with propensity score matched analysis.** *J Clin Med* 2020; **9:** 3703.
7. Lin J-W, Wang M-J, Yu H-Y, et al. **Comparing the survival between extracorporeal rescue and conventional resuscitation in adult in-hospital cardiac arrests: propensity analysis of three-year data.** *Resuscitation* 2010; **81:** 796–803.
8. Maekawa K, Tanno K, Hase M, Mori K, Asai Y. **Extracorporeal cardiopulmonary resuscitation for patients with out-of-hospital cardiac arrest of cardiac origin: a propensity-matched study and predictor analysis.** *Crit Care Med* 2013; **41:** 1186–96.
9. Okada Y, Komukai S, Irisawa T, Yamada T, Yoshiya K, Park C, Nishimura T, Ishibe T, Kobata H, Kiguchi T *et al*: **In-hospital extracorporeal cardiopulmonary resuscitation for patients with out-of-hospital cardiac arrest: an analysis by time-dependent propensity score matching using a nationwide database in Japan**. *Critical Care* 2023, **27**(1):442.
10. Patricio D, Peluso L, Brasseur A, et al. **Comparison of extracorporeal and conventional cardiopulmonary resuscitation: a retrospective propensity score matched study.** *Crit Care* 2019; **23:** 27.
11. Shin TG, Choi J-H, Jo IJ, et al. **Extracorporeal cardiopulmonary resuscitation in patients with inhospital cardiac arrest: a comparison with conventional cardiopulmonary resuscitation.** *Crit Care Med* 2011; **39:** 1–7.
12. Shin TG, Jo IJ, Sim MS, et al. **Two-year survival and neurological outcome of in-hospital cardiac arrest patients rescued by extracorporeal cardiopulmonary resuscitation.** *Int J Cardiol* 2013; **168:** 3424–30.
13. Suverein MM, Delnoij TSR, Lorusso R, et al. **Early extracorporeal CPR for refractory out-of-hospital cardiac arrest.** *N Engl J Med* 2023; **388:** 299–309.
14. Yannopoulos D, Bartos J, Raveendran G, et al. **Advanced reperfusion strategies for patients with out-of-hospital cardiac arrest and refractory ventricular fibrillation (ARREST): a phase 2, single centre, open-label, randomised controlled trial.** *Lancet* 2020; **396:** 1807–16.

# **Table S1a. Cochrane risk of bias tool 2.0 for randomised controlled trials**

| Study | Risk of bias sources | | | | | | Overall risk of bias |
| --- | --- | --- | --- | --- | --- | --- | --- |
|  | Randomisation | Deviations from intended intervention | Missing outcomes data | Outcomes measurement | Selection of reported results | Others |  |
| Belohlavek 2022 | Low risk | Low risk | Low risk | Low risk | Low risk | Low risk | Low risk |
| Suverein 2023 | Some concerns | Some concerns | Low risk | Low risk | Low risk | Low risk | Some concerns |
| Yannopoulos 2020 | Low risk | Low risk | Low risk | Low risk | Low risk | Low risk | Low risk |

# **Table S1b. Newcastle Ottawa Score for cohort studies**

| Study | Summary of risk of bias assessment | | | | | | | | |
| --- | --- | --- | --- | --- | --- | --- | --- | --- | --- |
|  | Representativeness of exposed cohort | Selection of non-exposed cohort | Ascertainment of exposure | Demonstration that outcome of interest not present at start of study | Comparability of cohorts on basis of design or analysis | Assessment of outcome | Follow up long enough for outcomes to occur | Adequacy of follow up of cohorts | Total score (out of 9) |
| Blumenstein 2016 | X | X | X | X | XX | X | X | X | 9 |
| Chen 2008 | X | X | X | X | XX | X | X | X | 9 |
| Choi 2023 | X | X | X | X | XX | X | X | X | 9 |
| Jeong 2022 | X | X | X | X | XX | X |  | X | 8 |
| Kim 2020 | X | X | X | X | XX | X | X | X | 9 |
| Lin 2010 | X | X | X | X | X | X | X | X | 8 |
| Maekawa 2013 | X | X | X | X | XX | X | X | X | 9 |
| Okada 2023 | X | X | X | X | XX | X | X | X | 9 |
| Patricio 2019 | X | X | X | X | X | X | X | X | 8 |
| Shin 2011/13 | X | X | X | X | XX | X | X | X | 9 |

#

# **Table S2. Grading of Recommendations, Assessment, Development, and Evaluations**

| **Certainty assessment** | | | | | | | **№ of patients** | | **Effect** | | **Certainty** | **Importance** |
| --- | --- | --- | --- | --- | --- | --- | --- | --- | --- | --- | --- | --- |
| **№ of cohorts** | **Study design** | **Risk of bias** | **Inconsistency** | **Indirectness** | **Imprecision** | **Other considerations** | **ECPR** | **CCPR** | **Relative (95% CI)** | **Absolute (95% CI)** |  |  |
| **In-hospital mortality (assessed with: odds ratio)** | | | | | | | | | | | | |
| 14 | 11 PSM, 3 RCTs | not serious | not serious | not serious | not serious | none | 4859/6336 (76.7%) | 6411/7712 (83.1%) | **OR 0.63** (0.50 to 0.79) | **75 fewer per 1,000** (120 fewer to 36 fewer) | ⨁⨁⨁⨁ High | CRITICAL |
| **Survival up to discharge/30 days with favourable neurological outcome (Cerebral performance category 1-2) (assessed with: odds ratio)** | | | | | | | | | | | | |
| 10 | 8 PSM, 2 RCT | not serious | not serious | not serious | not serious | none | 306/2391 (12.8%) | 331/3767 (8.8%) | **OR 1.57** (1.14 to 2.15) | **44 more per 1,000** (11 more to 84 more) | ⨁⨁⨁⨁ High | CRITICAL |
| **30-day survival (assessed with: odds ratio)** | | | | | | | | | | | | |
| 9 | 8 PSM, 1 RCT | not serious | not serious | not serious | not serious | none | 1377/5689 (24.2%) | 1030/5697 (18.1%) | **OR 1.70** (1.29 to 2.26) | **92 more per 1,000** (41 more to 152 more) | ⨁⨁⨁⨁ High | CRITICAL |

**CI:** confidence interval; **OR:** odds ratio, **PSM:** propensity-score matched study, **RCT:** randomised controlled trial

GRADE analysis was re-done for re-analysed outcomes only

# **Table S3:** Overall table summarising the re-analysed results, with forest plots and trial-sequential analyses graphs

| **Re-analysed outcome** | **Forest plot** | **Trial-sequential analysis** |
| --- | --- | --- |
| Overall mortality | **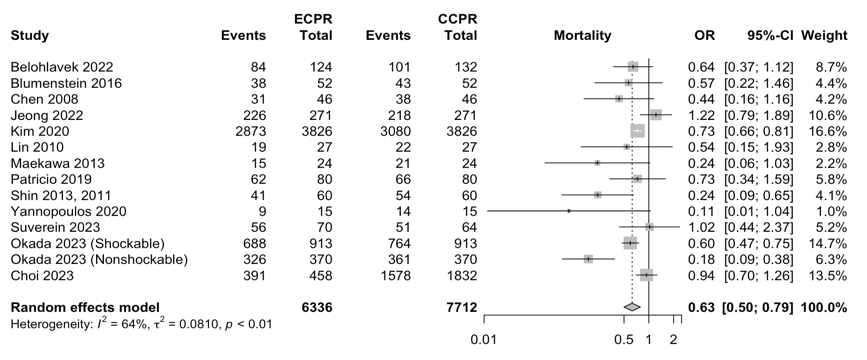** | **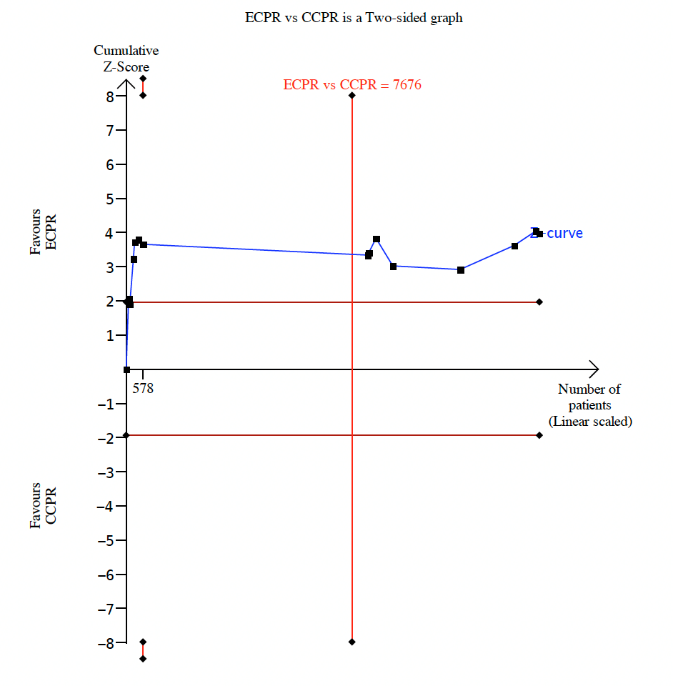** |
| PSM mortality | **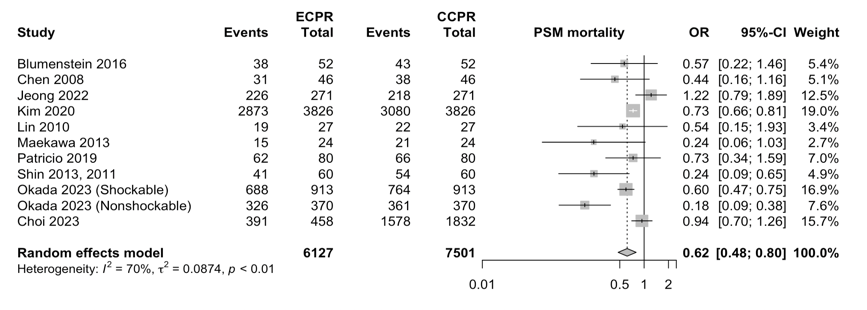** | 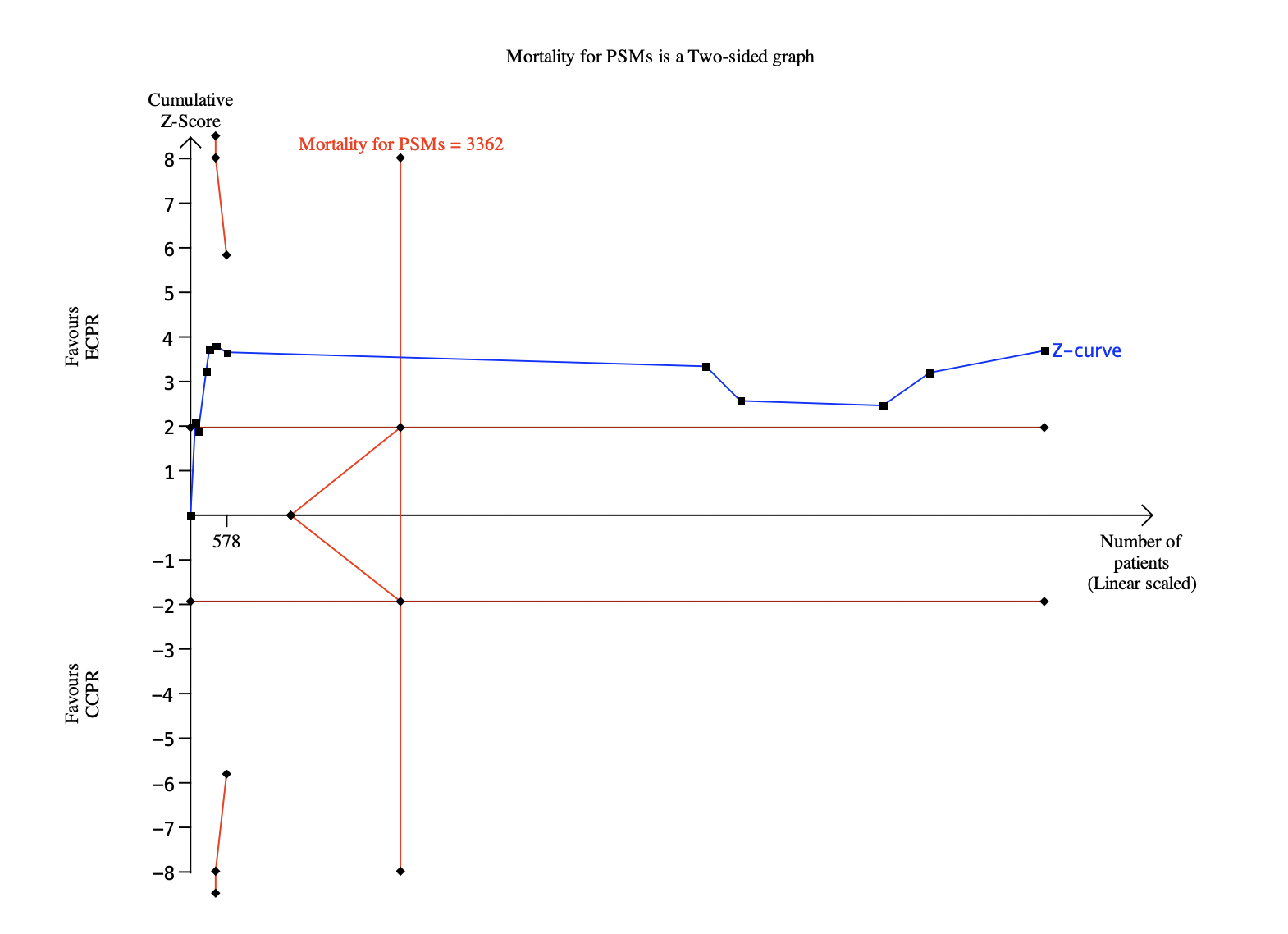 |
| OHCA mortality | **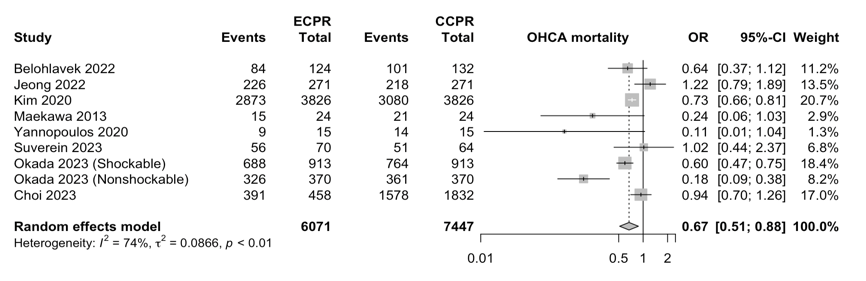** | 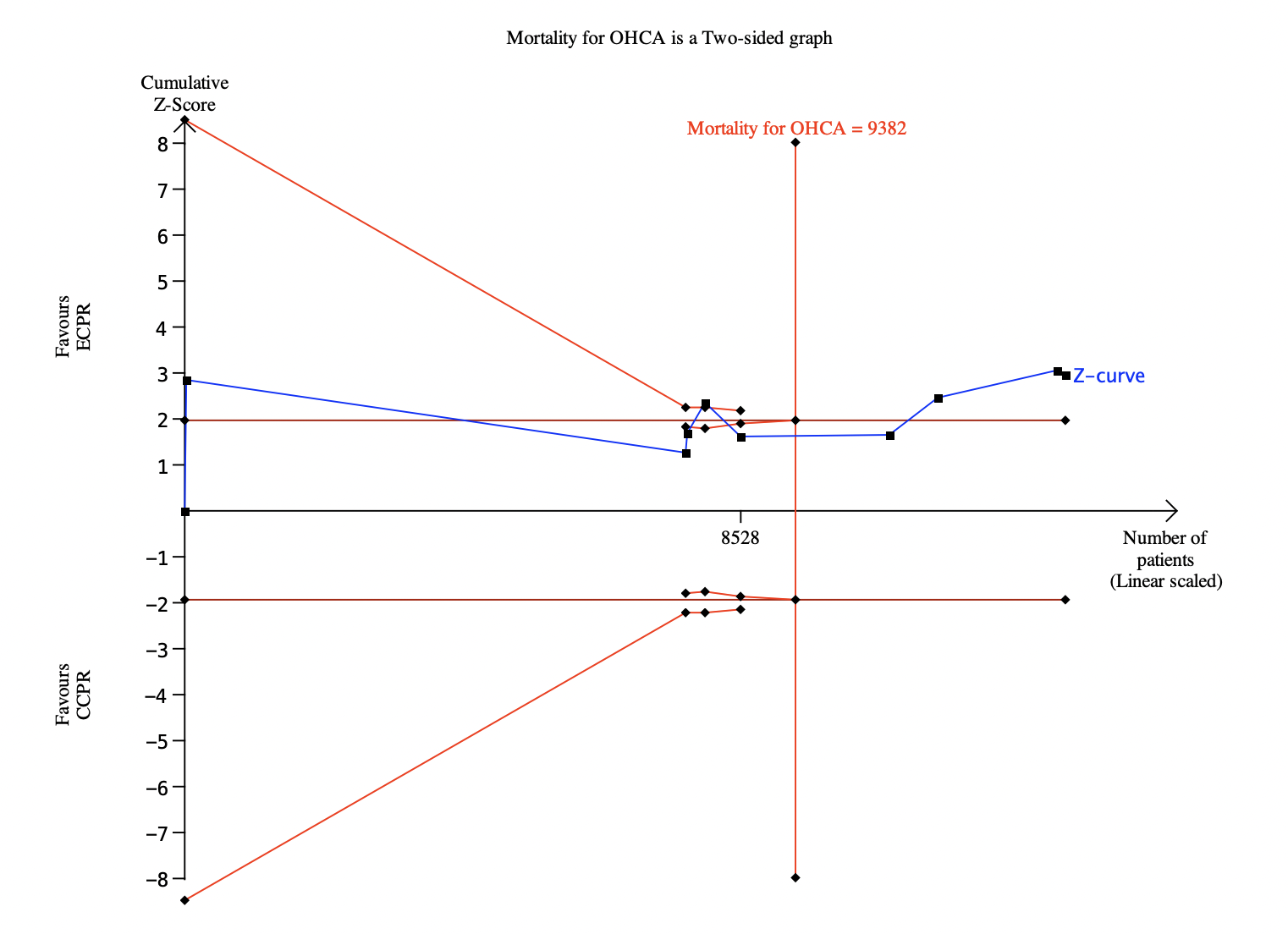 |
| 30-day survival | **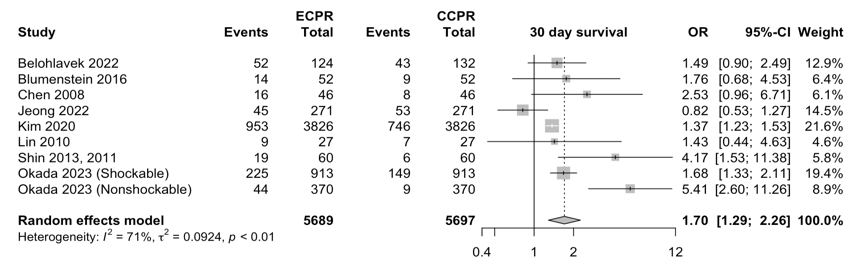** | 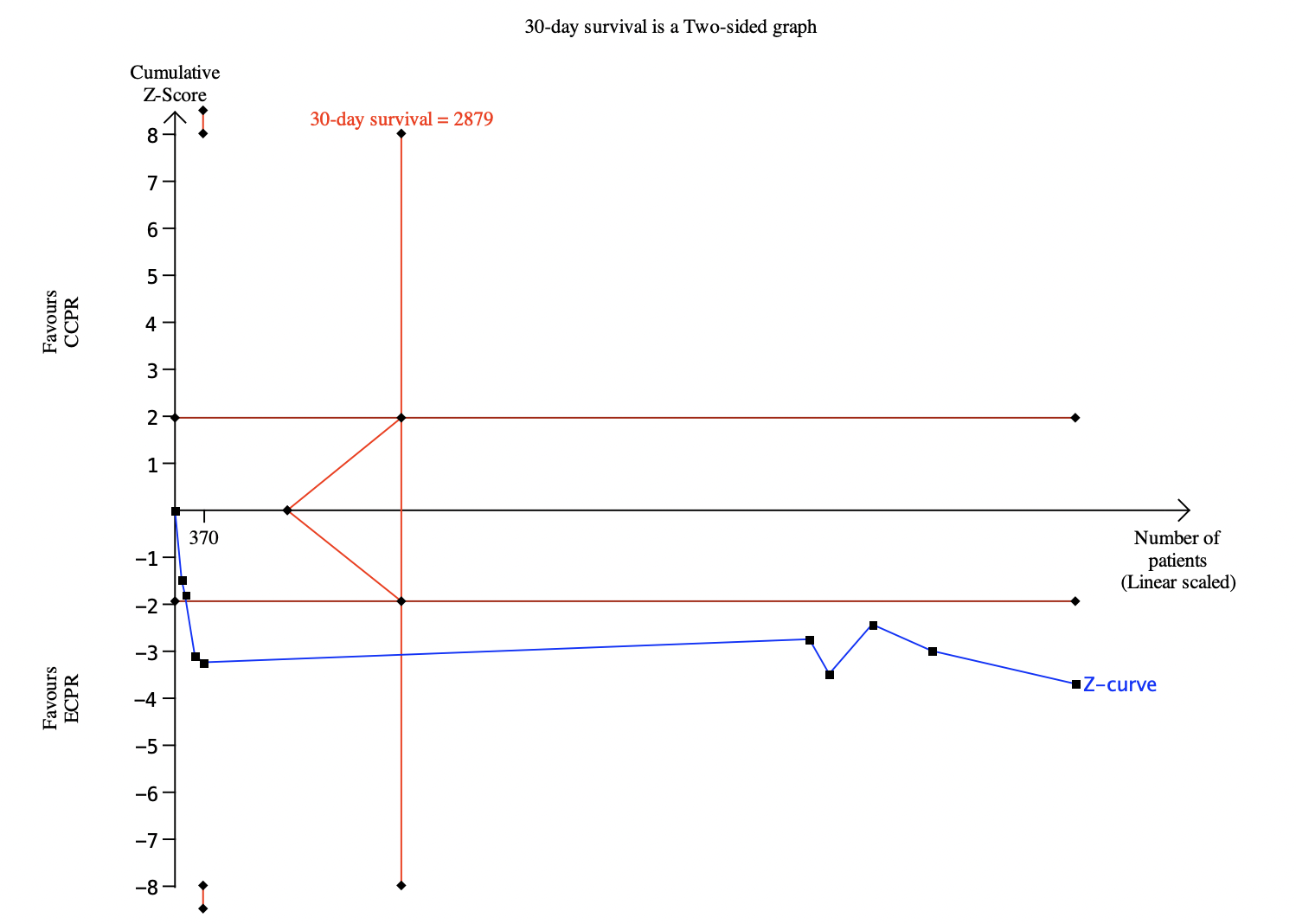 |
| Short-term favourable neurological outcome | **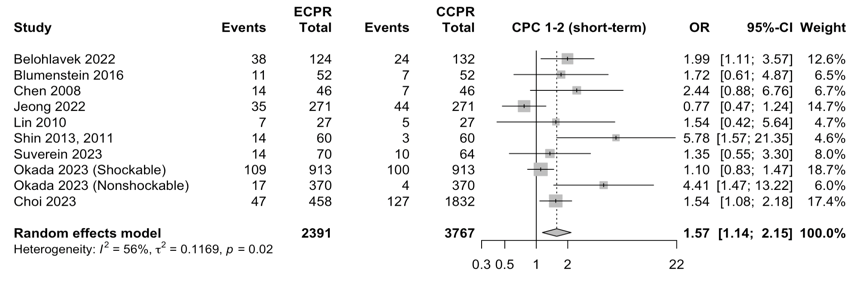** | 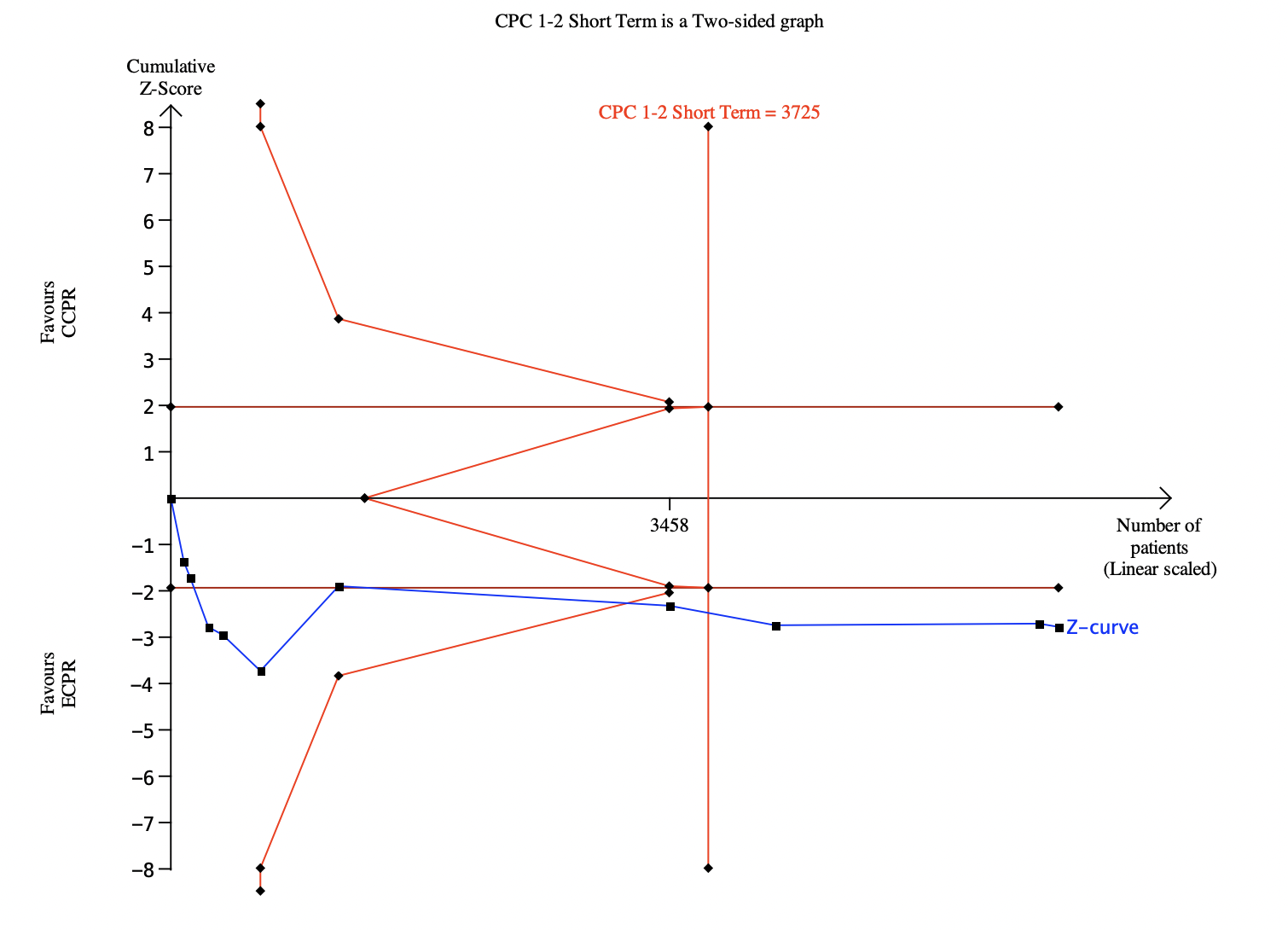 |
| **Additional subgroup analysis** | **Subgroup estimates** | **p-interaction** |
| Type of study | OHCA OR: 0.67, 95%-CI: 0.51 to 0.88  IHCA OR: 0.42, 95%-CI: 0.25 to 0.70 | 0.11 |
| Geographical region | OHCA OR: 0.67, 95%-CI: 0.51 to 0.88  Asia OR: 0.60, 95%-CI: 0.45 to 0.80  North America OR: 0.11, 95%-CI: 0.011 to 1.04 | 0.25 |
| Location of arrest | RCT OR: 0.65, 95%-CI: 0.32 to 1.34  PSM OR: 0.62, 95%-CI: 0.48 to 0.80 | 0.88 |
| Study quality | Low risk OR: 0.37, 95%-CI: 0.074 to 1.89  Some concerns OR: 1.02, 95%-CI: 0.44 to 2.37  NOS 8 OR: 0.98, 95%-CI: 0.64 to 1.50  NOS 9 OR: 0.55, 95%-CI: 0.41 to 0.73 | 0.092 |

## Table S4: Post-hoc sensitivity analysis

| Survival to hospital discharge | OR: 1.59, 95%-CI: 1.26 to 2.01. |
| --- | --- |
| Overall mortality (Studies with low risk on ROB2 and studies scoring 9 on NOS *1) | OR: 0.55, 95%-CI: 0.42 to 0.72 |

*1, In our study, we define “low bias risk” as receiving an assessment of low risk of bias for RCTs, and at least 8 out of 9 based on the Newcastle Ottawa Scale for propensity score-matched studies. All studies except the INCEPTION trial by Suverein et al. were assessed as having low bias risk.
